# Effects of high-fat diets on fetal growth in rodents: a systematic review

**DOI:** 10.1186/s12958-019-0482-y

**Published:** 2019-04-16

**Authors:** Julian K. Christians, Kendra I. Lennie, Lisa K. Wild, Raajan Garcha

**Affiliations:** Department of Biological Sciences, Simon Fraser University, 8888 University Drive, Burnaby, BC V5A 1S6 Canada

**Keywords:** Developmental origins, Fetal growth, Maternal nutrition, Obesity

## Abstract

**Background:**

Maternal nutrition during pregnancy has life-long consequences for offspring. However, the effects of maternal overnutrition and/ or obesity on fetal growth remain poorly understood, e.g., it is not clear why birthweight is increased in some obese pregnancies but not in others. Maternal obesity is frequently studied using rodents on high-fat diets, but effects on fetal growth are inconsistent. The purpose of this review is to identify factors that contribute to reduced or increased fetal growth in rodent models of maternal overnutrition.

**Methods:**

We searched Web of Science and screened 2173 abstracts and 328 full texts for studies that fed mice or rats diets providing ~ 45% or ~ 60% calories from fat for 3 weeks or more prior to pregnancy. We identified 36 papers matching the search criteria that reported birthweight or fetal weight.

**Results:**

Studies that fed 45% fat diets to mice or 60% fat diets to rats generally did not show effects on fetal growth. Feeding a 45% fat diet to rats generally reduced birth and fetal weight. Feeding mice a 60% fat diet for 4–9 weeks prior to pregnancy tended to increase in fetal growth, whereas feeding this diet for a longer period tended to reduce fetal growth.

**Conclusions:**

The high-fat diets used most often with rodents do not closely match Western diets and frequently reduce fetal growth, which is not a typical feature of obese human pregnancies. Adoption of standard protocols that more accurately mimic effects on fetal growth observed in obese human pregnancies will improve translational impact in this field.

**Electronic supplementary material:**

The online version of this article (10.1186/s12958-019-0482-y) contains supplementary material, which is available to authorized users.

## Background

The prenatal environment has far reaching effects on health throughout life [1]. Among the first observations of such effects were associations between birthweight and risk of cardiovascular disease and diabetes [2–5]. Initial studies found associations between low birthweight and adverse health outcomes later in life, whereas subsequent studies found that both very low and very high birthweight increased the risk of adult disease [6–8]. As a result of these associations, there is enormous interest in the long-term consequences of maternal overnutrition and/ or obesity during pregnancy [9]. However, the effects of maternal obesity on birthweight remain poorly understood.

Studies consistently report that obese women are at higher risk of having a large-for-gestational-age (LGA) baby [6, 10]. A higher frequency of LGA is due in part to obesity increasing the risk of gestational diabetes mellitus (GDM), which increases the risk of LGA [11]. However, maternal obesity also increases the risk of LGA in the absence of GDM [12]. Obese women are also at higher risk of hypertensive disorders of pregnancy such as preeclampsia, which is often associated with small-for-gestational-age (SGA) neonates [11]. This observation suggests that obese mothers might be at higher risk of SGA as well, but such an association is not observed consistently [13, 14]. There have been numerous reviews of the effects of dietary, exercise and lifestyle interventions on birthweight [13, 15–18], as well as associations between dietary intake and birthweight [13, 19–21]. However, these have not addressed why fetal growth is normal in many obese and diabetic pregnancies but altered in others [22].

Animal models are needed to better understand the effects of maternal obesity on fetal growth, and indeed numerous such studies have been performed, many using rodents fed high-fat diets (HFD). However, there is little standardization in this field, and the human phenotype being modeled is rarely defined more specifically than “obesity in pregnancy”. While there are numerous reviews of the effects of maternal overnutrition and/ or obesity on offspring glycemic control [23] and cardiovascular health [24, 25] in animal models, to our knowledge there has been no review of the effects of maternal HFD on birthweight in rodents. Rodents are born at a different developmental stage than humans, with birth in rodents corresponding to the end of the second trimester/ beginning of the third trimester in humans [26]. Nevertheless, hundreds of studies use rodent pregnancy as a model of human pregnancy, and thus it is necessary to consider to what extent rodent models mimic humans with respect to fetal growth; rodent birthweight is expected to provide a model of human fetal growth over the first two trimesters.

The purpose of this review is to identify factors (macronutrient composition, duration of diet, strain, etc.) that contribute to reduced or increased fetal growth in rodent models of maternal overnutrition. Such a review is needed to enable the development of more relevant and standardized animal models of human phenotypes. We sought to identify factors that are common among animal models that report an increase or decrease in birthweight, and to assess the uniformity of studies that use similar models. Our aim was not to perform a meta-analysis to assess whether, on average, there was an effect of HFD on birthweight (e.g., [27]). Rather, our goal was to review studies with different methodologies to determine whether some manipulations yielded consistent effects on birthweight.

## Methods

We followed the Preferred Reporting Items for Systematic Reviews and Meta-Analyses (PRISMA) guidelines [28].

### Data sources and search

**Fig. 1:**
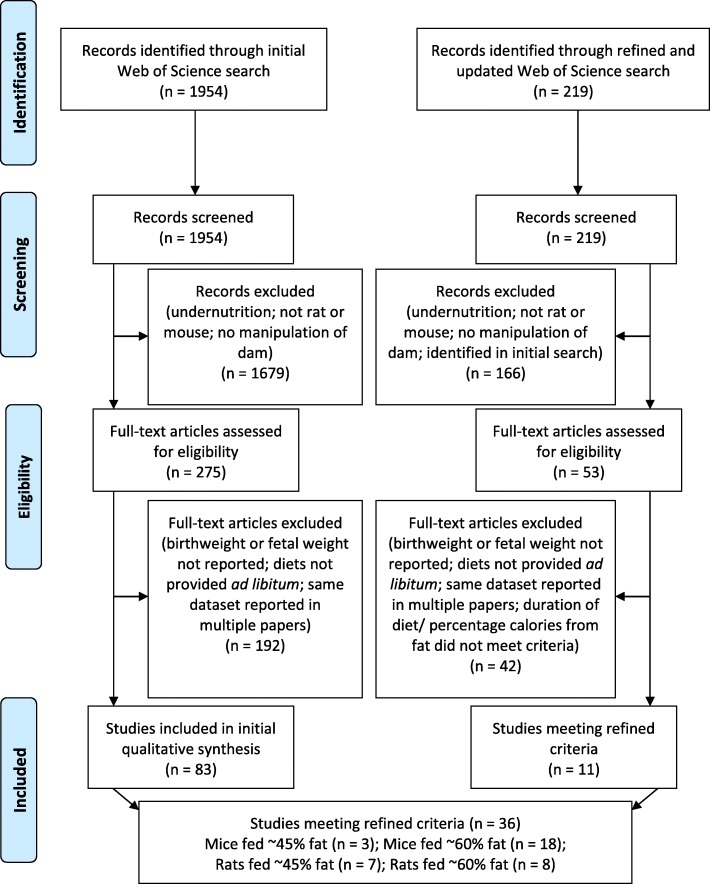
Flow diagram showing the initial and refined searches (figure template from [28])

The Web of Science database was searched using the terms: (maternal OR gestational) AND diet AND (birth OR fetal) AND weight AND (mouse OR rat). An initial search was conducted on July 12th, 2016, and an updated search was conducted on July 4, 2018 (the latter including only studies from 2016 to 2018). Figure 1 shows the selection process.

### Eligibility criteria

The participants, interventions, comparisons, outcomes, and study design (PICOS) criteria were as follows: participants were mice or rats; intervention was ad libitum access to a HFD during pregnancy (experimental diet may have also been provided prior to pregnancy, or may have been provided for only part of pregnancy); comparison was with a control diet provided ad libitum; outcome was birthweight (postnatal day 2 or earlier) or fetal weight of offspring from manipulated pregnancies (i.e., effects on subsequent generations were not included); study design was a controlled experiment. We included studies with any type of control diet that had a lower fat content than the intervention. Only studies published in English were included. Where more than one study appeared to describe the same dataset, only the first publication was included. Our updated, refined search added the following criteria: the HFD was provided for 3 weeks or more prior to pregnancy, and was either 44–45% calories from fat, or 57–62% calories from fat; cafeteria diets where animals were able to choose among food items were excluded.

### Study selection, data items and summary measures

In the initial search, 1954 titles and abstracts were screened and the full texts of 275 papers were reviewed. 83 studies reporting the birth or fetal weight of offspring were identified (Fig. 1). From each of these, the following variables were recorded: percentage fat content in the HFD, source of additional fat in the HFD (plant vs. animal), duration of diet prior to pregnancy, sample size per group, and age of dams at mating. Fat contents expressed as percentage of calories from fat were recorded if available. Our summary measure was whether the maternal HFD led to a statistically significant increase or decrease in birth and/ or fetal weight.

In the updated search using refined criteria, 219 titles and abstracts were screened, and after removing studies already identified in the initial search, 53 full texts were reviewed. This yielded 11 new papers reporting birth or fetal weight of offspring and, together with studies from the initial search, 36 papers were identified that matched the refined criteria (Fig. 1). In addition to the information collected for the initial search, we also recorded how maternal weight was affected by the experimental diet, whether maternal glucose tolerance was affected, whether litter size was affected, and more information regarding the control diet.

We assumed that animals were assigned to experimental groups at random, and therefore that there was little risk of bias within individual studies. However, we acknowledge that there was likely a publication bias across studies, whereby statistically significant effects were more likely to be reported.

## Results

The studies identified in the initial search, and the data extracted from each, are provided in Additional file 1: Table S1. Table 1 shows the number of studies finding an increase, decrease, or no effect on birthweight, aggregated by species, fat content, and duration of diet. More studies used rat than mouse as a model organism, and most used a HFD where the percentage of calories from fat was 45% or greater and included fat from animal sources (often lard) (Table 1, Additional file 1: Table S1). Among these studies, there were no consistent patterns with respect to effects on fetal growth. Increases and decreases were seen in studies of rats and mice, regardless of fat content, fat source or duration of diet prior to pregnancy. Although it might be expected that significant effects on birthweight would be found more often in studies with larger sample sizes, significant effects were approximately as frequent in studies with small sample sizes (< 10 dams per group) as in studies with moderate sample sizes (10–20 per group); few studies had greater than 20 dams per group (Additional file 1: Table S1).

**Table 1:** Summary of studies identified in initial search

Experimental protocol	Effect on fetal growth	Calories from fat				Total
		< 45%	45%	> 45%	Cafeteria	
Rats fed high-fat diet for 3 weeks or more prior to pregnancy	Decrease	0	4	2	4	10
			Nivoit et al. (2009) [29]; Hayes et al. (2012) [30]; Christante et al. (2013)^b^ [31]; Reynolds et al. (2014) [32]	Couvreur et al. (2011) [33]; Li et al. (2012)^b^ [34]	Akyol et al. (2009) [35]; Raipuria et al. (2015) [36]; Ramirez-Lopez et al. (2016) [37]; Sánchez-Blanco et al. (2016) [38]	
	No effect	3	2	8	3	16
		Del Prado et al. (1997) [39]; Caluwaerts et al. 2007 [40]; Nitert et al. (2013) [41]	Zambrano et al. (2010)^b^ [42]; Vega et al. (2015) ^b^ [43]	Shaw et al. (1997)^b^ [44]; Srinivasan et al. (2006) [45]; Ferezou-Viala et al. (2007) [46]; Gupta et al. (2009) [47]; Mitra et al. (2009) [48]; White et al. (2009) [49]; Guberman et al. (2013) [50]; Desai et al. (2014) [51]	Rolls and Rowe (1982) [52]; Chen et al. (2008) [53]; Ong and Muhlhausler (2011) [54]	
	Increase	0	1	2	1	4
			Song et al. (2015) [55]	Gaccioli et al. (2013)^b^ [56]; Mazzucco et al. (2013) [57]	Yang et al. (2015) [58]	
Rats fed high-fat diet for less than 3 weeks prior to pregnancy	Decrease	1	5	0	3	9
		Cerf et al. (2005) [59]	Mark et al. (2011) [60]; Smith et al. (2014) [61]; Cordero et al. (2015) [62]; Cunha et al. (2015)^b^ [63]; Segovia et al. (2015) [64]		Bayol et al. (2007) [65]; Zhang et al. (2011) [66]; Gugusheff et al. (2013) [67]	
	No effect	2	2	4	2	10
		Martin et al. (1987) [68]; Cerf et al. (2010)^a^ [69]	Yang et al. (2012) [70]; Tsoulis et al. (2016) [71]	Ebesh et al. (1999) [72]; Walker et al. (2008)^b^ [73]; Lin et al. (2011)^b^ [74]; de Oliveira Andrade et al. (2014) [75]	Rolls et al. (1984) [76]; Zhang et al. (2013) [77]	
	Increase	4	1	1	1	7
		Cerf et al. (2010)^a^ [69]; Ferro Cavalcante et al. (2013) [78]; Ferro Cavalcante et al. (2014) [79]; Cerf et al. (2015) [80]	Strakovsky et al. (2011) [81]	de Assis et al. (2006)^b^ [82]	Kjaergaard et al. (2014) [83]	
Total number of rat studies^a^		9^a^	15	17	14	55^a^
Mice fed high-fat diet for 3 weeks or more prior to pregnancy	Decrease	1	0	7	0	8
		Mayor et al. (2015) [84]		Niculescu and Lupu (2009) [85]; Bentham et al. (2010)^b^ [86]; King et al. (2013) [87]; King et al. (2013)^a^ [88]; Sasson et al. (2015) [89]; Edlow et al. (2016) [90]; Panchenko et al. (2016) [91]		
	No effect	0	2	4	0	6
			Lager et al. (2014)^b^ [92]; Umekawa et al. (2015) [93]	Liang et al. (2009) [94]; Bytautiene et al. (2011) [95]; King et al. (2013)^a^ [88]; Murabayashi et al. (2013) [96]		
	Increase	2	0	5	0	7
		Aye et al. (2015) [97]; Rosario et al. (2015) [98]		Masuyama and Hiramatsu (2012) [99]; Masuyama and Hiramatsu (2012) [100]; Dahlhoff et al. (2014) [101]; Masuyama and Hiramatsu (2014) [102]; Masuyama et al. (2015) [103]		
Mice fed high-fat diet for less than 3 weeks prior to pregnancy	Decrease	1	1	1	0	3
		Sferruzzi-Perri et al. (2013)^a^ [104]	Turdi et al. (2013) [105]	del Mar Plata et al. (2014) [106]		
	No effect	1	2	1	0	4
		Sferruzzi-Perri et al. (2013) ^a^ [104]	Luijten et al. (2013)^b^ [107]; Benatti et al. (2014) [108]	Volpato et al. (2012) [109]		
	Increase	0	1	1	0	2
			Ashino et al. (2012) [110]	Gregorio et al. (2013) [111]		
Total number of mouse studies^a^		4^a^	6	18^a^	0	28^a^

^a^Study is listed in the table twice because it included two time points or multiple fat contents with different results. The total number of studies per species counts each study only once

^b^Percentage of calories from fat was estimated based on energy density of diet, using values from two Research Diets (New Brunswick, NJ) diets (D12451: 45% fat by calories, 4.7 kcal/g, 24% fat by weight, and D12492: 60% fat by calories, 5.21 kcal/g, 35% fat by weight)

The diversity of experimental designs makes comparisons among studies difficult, but studies which include more than one experimental group are useful for examining specific factors that influence the effect on birthweight. One study found that feeding rats diets containing 20 or 30% fat (by energy) from the start of gestation increased birthweight compared with a diet of 10% fat, whereas a 40% fat diet did not [69]. Work by the same group found that a 40% fat diet during the first week of pregnancy reduced birthweight, whereas the same diet fed throughout pregnancy did not [59]. In contrast, other work by this group found that the weight of 20 day-old fetuses was increased by a 40% fat diet administered in third week of gestation, but not by the same diet throughout gestation [80]. Work by a different group found that a cafeteria diet fed to rats for 8 weeks prior to pregnancy reduced fetal weight at day 20, whereas the same diet fed from the start of pregnancy did not [35].

Effects of a maternal HFD may be consistent across gestational ages [89, 93, 101] but may vary through pregnancy. A diet high in fat and simple sugars fed to female mice from the beginning pregnancy reduced fetal weight at G15.5 but not G18.5 [104]. In contrast, a diet with a much higher fat content, and also high in simple sugars, fed to female mice for 12 weeks prior to pregnancy had no effect fetal weight at 14.5 but reduced fetal weight in day 18.5 male fetuses but not female fetuses [88]. Work by the same group found that the same protocol reduced birthweight in females but not males [87]. Inconsistencies in sex-specific effects may arise if the differences between males and females are not tested explicitly, e.g., using a sex by treatment interaction [112]. Studies by a single lab group using the same protocol may yield results that are similar [99, 100, 103] or divergent [66, 77].

Our initial survey did not reveal factors associated with maternal overnutrition that consistently led to increased or decreased fetal growth. We therefore focused more closely on a subset of studies with similar protocols and updated our search. Specifically, we focused on studies using HFDs containing 44–45% fat or 57–62% fat (by energy), as these were relatively numerous. We also restricted this refined search to studies that initiated experimental diets 3 weeks or more prior to pregnancy to include effects of maternal obesity rather than gestational overnutrition alone.

The studies identified in the refined search are summarized in Table 2, and the data extracted from each are provided in Additional file 1: Table S2. Our refined search found only 3 studies that fed mice a 45% fat diet, and all of these reported no effect on birth and/or fetal weight [93, 119, 120]. These studies had very similar protocols, using the same mouse strain (C57BL/6), the same HFD from the same manufacturer, and similar nutrient-matched 10% fat control diets (although these varied in sucrose content). The effect of a 58–60% diet on C57BL/6 mice was more variable, with 7 finding that HFD reduced birth and/or fetal weight [85, 87–91, 118], 3 finding no effect [94, 96, 121], and only one finding increased fetal weight [123], although the latter measured fetal weight much earlier than the others (at G12.5). Many of these studies used the same HFD from the same manufacturer, and some used nutrient-controls while others used chow as a control diet. However, even studies using identical HFD and control diets yielded divergent results (e.g., [85, 96] or [90, 123]). Among other mouse strains, the effects of a 60–62% fat diet increased birth and/or fetal weight in 6 studies [99–103, 122], although 5 of these were from the same group, and one study found no effect [95]. Among studies feeding 60% fat diets to mice, those finding an increase in fetal growth had fed the experimental diet for a shorter duration prior to pregnancy (4–9 weeks) than those reporting a reduction in fetal growth (9 weeks or more) (Additional file 1: Table S2). There was variability in the sucrose content of the control diets, but this was not associated with effects on birthweight (Additional file 1: Table S2).

**Table 2:** Summary of studies identified in the updated and refined search

Species	Effect on fetal growth	Calories from fat		Total
		~ 45%	~ 60%	
Rat	Decrease	6	1	7
		Nivoit et al. (2009) [29]; Hayes et al. (2012) [30]; Reynolds et al. (2014) [32]; Dodson et al. (2017) [113]; Huang et al. (2017) [114]; Ye et al. (2017) [115]	Yamada-Obara et al. (2016) [116]	
	No effect	0	7	7
			Srinivasan et al. (2006) [45]; Gupta et al. (2009) [47]; Mitra et al. (2009) [48]; White et al. (2009) [49]; Guberman et al. (2013) [50]; Desai et al. (2014) [51]; Lecoutre et al. (2016) [117]	
	Increase	1	0	1
		Song et al. (2015) [55]		
Total		7	8	15
Mouse	Decrease	0	7	7
			Niculescu and Lupu (2009) [85]; King et al. (2013) [87]; King et al. (2013)^a^ [88]; Sasson et al. (2015) [89]; Edlow et al. (2016) [90]; Panchenko et al. (2016) [91]; Bae-Gartz et al. (2016) [118]	
	No effect	3	5	8
		Umekawa et al. (2015) [93]; Chin et al. (2017) [119]; Jonscher et al. (2017) [120]	Liang et al. (2009) [94]; Bytautiene et al. (2011) [95]; King et al. (2013)^a^ [88]; Murabayashi et al. (2013) [96]; Connor et al. (2018) [121]	
	Increase	0	7	7
			Masuyama and Hiramatsu (2012) [99]; Masuyama and Hiramatsu (2012) [100]; Dahlhoff et al. (2014) [101]; Masuyama and Hiramatsu (2014) [102]; Masuyama et al. (2015) [103]; Masuyama et al. (2016) [122]; Nam et al. (2017) [123]	
Total^a^		3	18^a^	21^a^

^a^Study is listed in the table twice because it included two time points or multiple fat contents with different results. The total number of studies per species counts each study only once

Rats fed a 45% fat diet generally had offspring with reduced birth and/or fetal weights [29, 30, 32, 113–115], although the only study that used a nutrient-matched control diet showed an increase in birthweight [55]. In contrast, feeding rats a 57–60% fat diet generally had no effect on birth or fetal weight [45, 47–51, 117], although one study, which also provided fructose in the water of dams on the HFD, found a reduction in birthweight [116]. Variation between studies was not due to strain, as most used Sprague-Dawley rats, and discordant results were observed with this strain.

In both mice and rats fed diets of ~ 45% fat or ~ 60% fat, the HFD generally increased maternal mass prior to conception and impaired glucose tolerance, at least when these parameters were reported (Additional file 1: Table S2). In one case, impaired glucose tolerance was observed in HFD-fed females even though the control diet had a higher sucrose content [96]. Given that mice and rats gestate multiple fetuses per pregnancy, fetal growth might be influenced by effects on litter size, e.g., a reduction in litter size would be expected to increase the growth of individual fetuses. However, most studies that reported litter size found no effects of maternal HFD (Additional file 1: Table S2), and some found reductions in both litter size and the weight of individual offspring [30, 85, 87].

## Discussion

Rodents fed HFD are frequently used to study the effects of maternal obesity on offspring health, but we found few consistent effects of maternal HFD on fetal growth. The importance of these findings is not diminished by the fact that rodents are born at a different developmental stage than humans; a rodent model that decreases fetal growth and another that increases fetal growth cannot both model the same human phenotype. Our initial search identified studies with a variety of experimental protocols, and this revealed no clear patterns with respect to how the effects of maternal HFD depend on species, duration of diet, or diet composition. When we refined our focus to studies that fed dams diets containing ~ 45% fat or ~ 60% fat (by energy) for 3 weeks or more prior to pregnancy, we found more consistent results. Mice fed 45% fat diets and rats fed 60% fat diets generally did not show effects on fetal growth. Feeding a 45% fat diet to rats generally reduced birth and fetal weight. However, results were more variable in mice fed a 60% fat diet, with studies feeding the HFD for shorter periods prior to pregnancy (4–9 weeks) more likely to report an increase in fetal growth, whereas those feeding for a longer period were more likely to report a reduction in fetal growth.

Some variability among similar studies is not surprising. Thousands of studies have used rodents fed HFD to study obesity and/or diabetes (not specifically in pregnancy), and effects on weight gain and glycemic control have been inconsistent. Effects depend on diet composition, duration of diet, age and strain of animal, and in some cases vary among different experiments from the same lab [124]. The nature of the control diet is also important, since studies that use chow as controls may differ from a defined HFD in terms of protein source and fiber in addition to fat [124, 125].

A variety of models are needed given that there are a variety of diets and lifestyle characteristics associated with human obesity. However, the translation of the results of animal studies to clinical practice and policy development would benefit from some standardization of models and/or more explicit relation of the model to a particular human phenotype. Below, we discuss three key issues to consider in the design of experiments to examine the long-term consequences of maternal overnutrition and/ or obesity, and provide recommendations for standardized models.

### Experimental diets and controls

The percentage of energy from fat in US diets is estimated to be in the range of 30–40% in obese and normal weight individuals [124, 126, 127], and therefore experimental diets with much more than 40% of energy from fat may provide weaker models of human pathophysiology. Similarly, the median percentage of calories from protein in US diets is 14% [128], whereas many of the studies that we identified used purified HFDs that were 20% protein, although some attempted to mimic the protein content of Western diets [113, 129]. Thus, while many experimental diets are successful in increasing maternal pre-pregnancy weight and/ or impairing glucose metabolism, the resulting obesity may not accurately reflect common human phenotypes. To facilitate putting experimental diets into a human context, studies should be required to report the macronutrient composition of experimental and control diets as a percentage of total energy, rather than only reporting contents by weight and/ or the overall energy density.

Although we focused on studies that provided a HFD ad libitum for a specific period prior to and/or during pregnancy, we acknowledge that other experimental approaches offer advantages for studying the effects of maternal obesity. Embryo transfers have been used to distinguish the effects of pre-gestational and gestational exposure to maternal obesity [89]. Shankar et al. [130] fed liquid diets to rats by intragastric cannulation to induce obesity by controlling the number of calories provided. A contrasting approach is to control food intake to keep energy consumption similar and vary only the macronutrient composition of the maternal diet [131]. To ensure that the HFD induces maternal obesity, some studies mate HFD females when their body weight has increased by a certain amount, and compare these with age-matched females on a control diet. In mice, this approach increased fetal weight in two studies [97, 98], had no effect in another [132], and decreased fetal weight in a fourth [90]. Ye et al. [115] fed rats a HFD and selected those with the greatest weight gain (susceptible to diet-induced obesity) and those with the lowest weight gain (resistant to diet-induced obesity) and found that fetal weight was reduced in susceptible dams but not in resistant dams.

### Effects on birthweight

It is concerning that a substantial number of studies found that a maternal HFD reduced fetal growth, because this is not a typical feature of obese pregnancy in humans. Although some reviews suggest that obese mothers are at increased risk of delivering small infants, closer scrutiny of the primary literature cited does not support such claims. To the contrary, some studies have found that obesity reduces the risk of delivering a small-for-gestational age or low-birthweight baby [133–136]. Obesity increases the risk of preeclampsia, and preeclampsia is often associated with intrauterine growth restriction [11, 137] but it does not necessarily follow that obesity increases the risk of intrauterine growth restriction. Studies reporting increased risk of preeclampsia in obese pregnancies generally do not find associations between obesity and IUGR [138, 139]. We know of only one study that found an increased risk of having a small-for-gestational-age infant among obese women, and this was found only in association with a body mass index (BMI) over 40, and not with a BMI of 29–40 [140]. The association was not significant after removing women with preeclampsia [140], suggesting that obesity specifically increased the risk of growth restriction in preeclamptic women. Thus, apart from this study, there is little evidence that human obesity is associated with fetal growth restriction. Therefore, experimental approaches that result in reduced fetal growth are less relevant as models of obesity and overnutrition in humans, and it may be useful to avoid the protocols that we have identified as consistently reducing fetal growth. Based on developmental milestones, rodent birthweight only provides a model of human fetal growth up to the end of the second trimester. However, it is unlikely that a rodent model with reduced birthweight is a good model for an obese human pregnancy with an increased risk of high birthweight, unless it can be shown that human obesity results in growth restriction early in pregnancy that recovers in the last trimester. Experimental approaches that increase fetal growth are suitable for modeling high birthweights, which occurs at higher frequency in obese pregnancies [6, 10]. Mating HFD mice when their weight had increased by 25% led to higher fetal weight in two studies [97, 98] but had no effect in another [132]. While the solid component of the HFD was 41% fat in these studies, the provision of a sucrose solution with the HFD would have reduced the percentage of calories from fat (and protein), perhaps to levels more typical of Western diets. Protocols with no effect on fetal growth may be useful for studying effects of maternal obesity on offspring health that occur independently of birthweight [141].

### Species and strain

As model species, rats and mice offer different strengths and weaknesses. The larger size of rats facilitates various physiological manipulations and measurements, while mice are more amenable to genetic manipulation, and their smaller size reduces cost per animal [142]. Rat strains are generally outbred (Wistar, Sprague Dawley, Long-Evans) while the most commonly used mouse strains (e.g., C57BL/6) are inbred [143]. While inbred strains reduce variability and thereby increase the power to detect certain effects, outbred strains may provide a better model of the genetic heterogeneity of human populations [25], and may also be of greater relevance for effects dependent on the immune system [142].

In a recent meta-analysis, maternal HFD tended to increase birthweight in mice and decrease birthweight in rats, although species and strain contributed relatively little to between-study variability in the metabolic effects of maternal HFD [27]. Strains of mice differ in their susceptibility to the metabolic effects of HFD [144], but it is not necessarily the case that more susceptible strains are better models; more resistant strains may offer the opportunity to study differences between obesity-susceptible and obesity-resistant individuals (e.g., [115]). Glucose metabolism even differs between substrains of C57BL/6 [145], which underscores the importance of reporting the substrain, e.g., C57BL/6 J vs. C57BL/6 N.

While mice and rats are frequently used to investigate the effects of maternal nutrition, these species are born at a substantially different developmental stage than humans and generally have multiple fetuses per pregnancy. In these and some other respects, other rodent models such as guinea pigs offer advantages over mice and rats [146]. A more complete consideration of species selection is provided elsewhere [142, 147]. As with the diet protocol, the rationale for the choice of species and the specific situation it attempts to model should be described explicitly [142]. Nevertheless, there are broad similarities in the phenotypes of offspring exposed to maternal HFD between mice, rats and non-human primates [143].

## Conclusions

The effects of HFD during pregnancy have been examined with a diverse array of experimental protocols yielding few clear patterns with respect to approaches that increase or decrease fetal growth. However, studies with similar protocols using the same strain of animal yield more consistent results. Even so, the HFD used most frequently with rodents do not closely match Western diets, the former being higher in fat and protein. Perhaps for this reason, many studies find that a maternal HFD reduces fetal growth in rodents, which is not a typical feature of obese human pregnancies. The present review has identified experimental approaches that increase birthweight or have no effect, and the adoption and standardization of such protocols will improve the translational impact of research into the effects of maternal overnutrition on offspring health.

## Additional file

Additional file 1: **Table S1.** Studies identified in the initial search; **Table S2.** Studies identified in the refined search. (XLSX 32 kb)

## Abbreviations

BMI: Body mass index
GDM: Gestational diabetes mellitus
HFD: High fat diet
LGA: Large-for-gestational-age
